# The actual and future role of molecular tests in thyroid pathology

**DOI:** 10.1007/s00428-025-04334-9

**Published:** 2025-11-17

**Authors:** Matthias S. Dettmer

**Affiliations:** 1 Klinikum Stuttgart, Institute of Pathology, Katharinenhospital, Kriegsbergstr. 60, Stuttgart, D-70174 Germany; 2 Institute of Tissue Medicine and Pathology, University of Bern, Bern, Switzerland

**Keywords:** Fine-needle aspiration cytology (FNAC), Thyroid nodules, Thyroid pathology, Molecular thyroid pathology, Molecular diagnostics

## Abstract

Thyroid nodules represent a common clinical challenge, with 20–30% of fine-needle aspiration biopsies yielding indeterminate cytology results that complicate management decisions. While fine-needle aspiration cytology (FNAC) remains the gold standard for initial evaluation, up to 30% of cases produce indeterminate results, often leading to unnecessary diagnostic surgeries. This comprehensive review examines the transformative role of molecular diagnostics in thyroid pathology, focusing on their clinical utility, prognostic implications, and future directions. Molecular testing platforms, including Afirma GSC, ThyroSeq v3, and ThyroidPrint, have transformed the management of indeterminate thyroid nodules through gene expression profiling, mutation analysis, and microRNA signatures. The third-generation tests exhibit high sensitivity (91–100%) and negative predictive values (90–100%), thereby enabling surgical avoidance rates of 50.3–68.6% for patients with indeterminate cytology. The platforms employ both “rule-out” strategies (high sensitivity/NPV) and “rule-in” approaches (high specificity/PPV) to guide clinical decision-making. The paradigm of classifying thyroid tumors based on BRAF-like and RAS-like molecular profiles is becoming increasingly entrenched in clinical and diagnostic practice, affording pathologists and clinicians the ability to render diagnoses that are both more precise and reproducible. Within this molecular framework, the identification of markers such as TERT promoter and TP53 mutations, along with gene fusions, provides not only refined prognostic information but also facilitates the selection of patients for targeted therapeutic regimens including BRAF/MEK inhibitors and RET inhibitors. Nevertheless, the implementation of these advances is not without its impediments. The field continues to grapple with platform heterogeneity, economic constraints, and geographic disparities affecting access to comprehensive molecular diagnostics—factors that necessitate ongoing efforts to standardize testing and expand global availability. The future of this field is marked by several key developments, including the expansion of next-generation sequencing, the advancement of liquid biopsy technologies, the integration of artificial intelligence, and the adoption of multi-omic approaches. International guidelines are increasingly recommending molecular testing for advanced thyroid cancers and indeterminate nodules. These guidelines emphasize the need for standardized protocols and equitable access to such testing. Molecular diagnostics should be embraced as complementary tools within multidisciplinary care to optimize patient outcomes while reducing unnecessary interventions in thyroid nodule management.

## Introduction

Thyroid nodules are a common clinical finding that occur in a significant proportion of the population over the course of a lifetime. While most nodules are benign, distinguishing between benign and malignant lesions remains a diagnostic challenge, particularly due to the low incidence of aggressive thyroid carcinoma in the general population. Fine-needle aspiration cytology (FNAC) is the gold standard for initial investigation, but in up to 30% of cases, the results are indeterminate, often requiring further diagnostic measures and sometimes unnecessary diagnostic surgery [4, 38, 56]

Advances in molecular diagnostics have significantly changed the landscape of thyroid pathology. Over the past decade, integrated molecular testing platforms such as Afirma GSC and ThyroSeq v3 have been further developed, providing robust tools for risk stratification in cytologically indeterminate nodules. These platforms analyze a broad spectrum of genetic alterations, including mutations, fusions and gene expression profiles, improving diagnostic accuracy and enabling personalized management strategies[3, 4, 10, 38, 66, 73]

Recent updates to international guidelines from organizations such as the European Thyroid Association (ETA), the American Thyroid Association (ATA), the National Comprehensive Cancer Network (NCCN), and expert recommendations have supported the integration of molecular data into clinical practice. These guidelines now recommend molecular testing as a diagnostic tool for indeterminate thyroid nodules. Additionally, molecular profiling aids in diagnosis and provides valuable prognostic information, as well as identifying targets for targeted therapies in advanced or recurrent thyroid cancer [3, 9, 10, 42, 45, 56, 61, 73]. Despite these advances, challenges remain, including variability in test performance, cost, and global accessibility disparities.

The paradigm of prioritizing molecular information over histological evaluation is not entirely novel. In fact, it has long been embedded within certain domains of pathology, most prominently hematopathology and neuropathology, where molecular findings constitute a diagnostic cornerstone. For practitioners in these fields encompassing clinicians and pathologists, a return to an era of reliance solely on the speculative interpretation of subtle morphological features would be inconceivable. Crucially, however, the added value of molecular approaches is realized only when molecularly defined groups or entities can be delineated in clinically relevant and ideally also morphologically coherent terms.

As the field continues to evolve, critically assessing the current evidence and anticipating future directions in molecular diagnostics for thyroid pathology is essential. This review aims to provide a comprehensive overview of the current and future roles of molecular tests in thyroid pathology. It will highlight their clinical utility, as well as their diagnostic, prognostic, and therapeutic implications. Additionally, it will address the challenges and opportunities that lie ahead [11, 35, 61].

## Diagnostic of thyroid nodules

### Conventional diagnostic approaches—a pathologists perspective

#### Fine-needle aspiration cytology (FNAC)

FNAC is the preferred initial diagnostic test for thyroid nodules due to its high accuracy, minimal invasiveness, and cost-effectiveness. Studies consistently demonstrate high sensitivity and specificity for FNAC, with diagnostic accuracy typically exceeding 90% in experienced hands [35, 67, 69]. While the Bethesda System for Reporting Thyroid Cytopathology provides a standardized framework for interpreting results, indeterminate categories (e.g., AUS/FLUS and follicular neoplasm) remain a diagnostic challenge and often necessitate further evaluation or surgery [59].

#### Histopathology

A definitive diagnosis of thyroid nodules requires a histopathological examination. Surgical resection and subsequent histopathology enable the evaluation of tissue architecture, capsular and vascular invasion, and the identification of characteristics that cannot be reliably diagnosed by cytology alone [8, 59].

#### Intraoperative frozen section

Intraoperative frozen sectioning (FS) is sometimes used to provide immediate diagnostic information during thyroid surgery. However, its usefulness is limited, particularly for follicular-patterned lesions. Distinguishing between adenoma and carcinoma often requires permanent sectioning to evaluate capsular and vascular invasion. Several studies report that FS has high specificity but variable sensitivity, particularly for follicular neoplasms [41, 63]. Meta-analyses confirm that FS demonstrates only moderate diagnostic performance in follicular neoplasms (sensitivity 43%, specificity 100%), leading some institutions to discourage its routine use, as it rarely changes surgical plans and can result in unnecessary delays and costs[32, 36, 63].

### The need for molecular diagnostics

#### Prevalence and impact of indeterminate nodules

Approximately 20–30% of thyroid nodules that undergo fine-needle aspiration biopsy (FNAB) yield indeterminate cytology results. These results most often fall into Bethesda categories III (AUS/FLUS) and IV (suspicious for follicular neoplasm)[67]. This substantial proportion of indeterminate nodules poses a persistent diagnostic challenge because the risk of malignancy in these categories ranges from 10 to 40%. This uncertainty often leads to unnecessary surgeries for diagnosis, with malignancy confirmed in only a minority of excised nodules [4, 56].

#### Clinical and economic consequences

The diagnostic uncertainty associated with indeterminate nodules creates significant clinical and economic challenges. Unnecessary thyroid surgeries expose patients to risks such as hypoparathyroidism and recurrent laryngeal nerve injury, while also increasing healthcare costs [4]. The psychological impact on patients awaiting a definitive diagnosis is significant. Anxiety and a reduced quality of life are commonly reported during this process.

#### The role of precision medicine

Thanks to advances in molecular diagnostics, new tools are available for risk stratification and personalized management of thyroid nodules. Molecular tests can more accurately distinguish between benign and malignant nodules by analyzing specific genetic alterations, gene expression profiles, and microRNA signatures, particularly in cases of indeterminate cytology [4, 56]. This approach aligns with the broader trend of precision medicine, which aims to optimize patient outcomes by providing personalized diagnostic and therapeutic strategies[4, 56].

#### Integration with clinical guidelines

Recent updates to international guidelines and expert recommendations, including those from the American Thyroid Association (ATA) and the European Thyroid Association (ETA), now recommend the use of molecular testing for indeterminate thyroid nodules and advanced thyroid cancers [9, 11, 35, 42, 45, 61]. These guidelines emphasize the potential of molecular diagnostics to reduce unnecessary surgeries and improve the accuracy of preoperative risk assessment [11, 35, 61]. Molecular reporting has become increasingly comprehensive, incorporating clinical information such as nodule size and Bethesda category. This integration facilitates informed discussions between physicians and patients regarding nodule management [4, 11, 35, 61, 69].

## Molecular pathology

### Types of molecular tests

#### Gene expression classifiers (e.g., Afirma GSC)

Gene expression classifiers are analytical tools that evaluate the expression patterns of multiple genes to facilitate the distinction between benign and malignant thyroid nodules. The Afirma Genomic Sequencing Classifier (GSC) is one of the most widely used tests in this category. It employs RNA whole-transcriptome sequencing and machine learning to evaluate indeterminate thyroid nodules, providing a comprehensive genomic assessment that can inform clinical decisions [29, 58]. The Afirma GSC has demonstrated a higher benign call rate and improved specificity compared to its predecessor, the Gene Expression Classifier (GEC), thereby reducing unnecessary surgeries for patients with indeterminate cytology [46, 62, 72].

#### Gene mutation panels (e.g., ThyroSeq, ThyGeNEXT/ThyraMIR)

NGS has revolutionized the molecular diagnosis of thyroid nodules by enabling simultaneous analysis of multiple genes and gene fusions, significantly improving diagnostic sensitivity and specificity compared to single-gene assays. Recent studies demonstrate that NGS-based panels, including up to 17 or more genes, achieve sensitivities and specificities above 85% for indeterminate thyroid nodules, markedly enhancing the ability to stratify risk and guide surgical decisions [15].

ThyroSeq v1–3, for instance, utilizes next-generation sequencing to identify mutations in genes such as *BRAF*, *RAS*, *TERT*, and gene fusions like *RET-PTC* and *PAX8-PPARG*, which exhibit high specificity for malignancy [49–51]. Conversely, ThyGeNEXT/ThyraMIR is a multiplatform test that integrates mutation detection with microRNA (miRNA) expression profiling to enhance diagnostic accuracy. The value of these panels is particularly evident in cases of indeterminate cytology, where they provide actionable information that facilitates the development of management strategies and reduces the frequency of unnecessary surgical procedures [19, 64, 69].

Expanded panels now routinely include not only common mutations such as *BRAF* and *RAS* but also *TERT* promoter, *EIF1AX*, and gene fusions involving *RET*, *NTRK*, and *ALK*, providing both diagnostic and therapeutic information [15]. These advances allow clinicians to tailor management strategies more precisely and identify candidates for targeted therapies, especially in advanced or refractory cases [11, 35, 45, 61].

#### MicroRNA expression profiling

MicroRNA (miRNA) profiling has emerged as a promising adjunct to mutation and gene expression analysis. miRNAs are small non-coding RNAs that regulate gene expression and are frequently deregulated in thyroid cancer [13, 25]. Distinct miRNA signatures have been identified for different thyroid tumor types, including papillary, follicular, poorly differentiated, and oncocytic thyroid carcinomas [22, 23, 25]. Conventional and oncocytic follicular thyroid carcinomas have been shown to have unique microRNA (miRNA) profiles. Specific miRNAs, such as miR-885-5p, have been found to be strongly upregulated in oncocytic follicular carcinoma. This finding suggests that these specific miRNAs may serve as potential diagnostic markers for this particular subtype [23]. miRNA profiling can be performed on fine-needle aspiration samples. This procedure has been integrated into commercial tests, such as ThyraMIR, with the objective of improving diagnostic accuracy for indeterminate nodules [19, 64, 69].

### Clinical utility in indeterminate nodules

#### Bethesda III/IV nodules: risk stratification

The primary diagnostic challenge in thyroid nodule evaluation arises with Bethesda III (atypia of undetermined significance/follicular lesion of undetermined significance, AUS/FLUS) and Bethesda IV (suspicious for follicular neoplasm) nodules, collectively termed indeterminate thyroid nodules (ITNs). These categories account for approximately 20–25% of thyroid nodule aspirates, with malignancy risk ranging from 6 to 40%, depending on institutional practices and the classification of noninvasive follicular thyroid neoplasm with papillary-like nuclear features (NIFTP) [16, 18, 35, 37]. Molecular testing has significantly improved risk stratification by providing additional data to guide management decisions and reduce unnecessary surgeries [16, 37].

#### Rule-in and rule-out strategies

Molecular tests are broadly categorized into “rule-in” and “rule-out” strategies:

- Rule-out tests (e.g., Afirma GSC, ThyroidPrint®) are designed to identify nodules with a low likelihood of malignancy, thereby supporting nonoperative management for patients with negative results (Table 1). These tests aim for high sensitivity and negative predictive value (NPV), allowing clinicians to confidently avoid surgery in most benign cases [53, 56].

**Table 1:** Comparison of Major Platforms

Platform	Sensitivity (%)	Specificity (%)	NPV (%)	PPV (%)	Strategy
Afirma GSC	90–100	65–85	94–100	50–60	Rule-out
ThyroSeq v3	94–97	50–82	93–99	60–66	Rule-in/Rule-out hybrid
ThyroidPrint®	90–91	87–89	94–96	71–82	Rule-out
- Rule-in tests (e.g., ThyroSeq, ThyGeNEXT) seek to identify nodules with a high likelihood of malignancy, supporting surgical intervention when positive (Table 1). These tests aim for high specificity and positive predictive value (PPV), helping to prioritize surgery for patients with a high risk of cancer [19, 51, 56].

#### Performance metrics (sensitivity, specificity, NPV, PPV)

The clinical utility of molecular tests is best assessed by their diagnostic performance metrics:

- Sensitivity: Proportion of malignant nodules correctly identified (true positive rate).
- Specificity: Proportion of benign nodules correctly identified (true negative rate).
- Negative predictive value (NPV): Likelihood that a negative test result truly indicates a benign nodule.
- Positive predictive value (PPV): Likelihood that a positive test result truly indicates a malignant nodule.

Recent validation studies and meta-analyses of major platforms demonstrate the following:

- Afirma GSC: Sensitivity 91–100%, specificity 43–77%, NPV 90–100%, PPV 47–63% in real-world cohorts [37, 56].
- ThyroSeq v3: Sensitivity 94–97%, specificity 50–83%, NPV 92–99%, PPV 64–70% across independent studies [37, 56].
- ThyroidPrint® (rule-out): Sensitivity and NPV are high, supporting its use as a rule-out test in ITNs [53, 70].

### Impact on clinical decision-making

#### Reduction in unnecessary surgeries

Meta-analyses and multi-center studies consistently demonstrate that molecular testing is cost-effective, reduces unnecessary surgeries, frees up valuable resources in the operating room, improves diagnostic accuracy in ITNs, and lowers the risk for patients with high sensitivity and NPV across platforms [17, 28, 37, 39, 51, 53, 70]. For example, pooled data from 31 studies and 4,464 nodules show that molecular platforms such as ThyroSeq, Afirma, and ThyGenX/ThyraMIR achieve surgical avoidance rates ranging from 50.3 to 68.6% with ThyGenX/ThyraMIR demonstrating the highest rate (68.6%) and lowest heterogeneity, suggesting robust and consistent performance across institutions [17].

#### Guiding extent of surgery and implications for patient counseling and follow-up

Molecular testing has become essential in guiding the surgical management of thyroid nodules, particularly those with indeterminate cytology. High-risk mutations, such as *BRAF* V600E, *TERT* promoter mutations, and *RET/PTC* fusions, often prompt total thyroidectomy due to their association with aggressive disease and multifocality. Conversely, low-risk or negative molecular profiles support a more conservative approach, such as lobectomy or active surveillance [44, 60]. This stratification helps minimize unnecessary extensive surgeries while optimizing patient outcomes. Additionally, molecular testing enhances patient counseling by clarifying malignancy risk and informs individualized follow-up strategies, allowing closer monitoring of high-risk cases and less intensive surveillance for low-risk patients [17, 28].

### Molecular markers

#### *BRAF* V600E and other single-gene assays

The *BRAF* V600E mutation is the most prevalent genetic alteration in papillary thyroid carcinoma (PTC). Despite its frequency, recent meta-analyses and large cohort studies demonstrate that *BRAF* V600E does not independently predict overall survival (OS) or disease-free survival (DFS) in follicular-derived thyroid cancers. Specifically, pooled analyses show no statistically significant effect of *BRAF* mutations on OS (HR = 1.11, 95% CI 0.66–1.88) or DFS (HR = 1.23, 95% CI 0.66–2.29)[47]. While *BRAF* V600E is associated with certain aggressive histological subtypes and can co-occur with other high-risk mutations, its prognostic value is limited when considered in isolation. Nevertheless, *BRAF* V600E remains important for diagnostic confirmation and has direct implications for targeted therapy in advanced disease [1].

Other single-gene assays may target mutations in *RAS*, *TERT* or gene rearrangements such as *RET-PTC* and *PAX8-PPARG*. These assays are often used when a specific mutation is suspected based on clinical or cytological features, and they provide rapid, targeted information to guide management [12, 26].

#### *TERT* promoter and *TP53* mutations

In contrast to *BRAF*, *TERT* promoter mutations are robustly associated with poor prognosis in thyroid cancer [27, 47]. Recent systematic reviews and meta-analyses confirm that *TERT* promoter mutations confer a significantly increased risk of both worse OS (HR = 1.90, 95% CI 1.17–4.47) and DFS (HR = 2.76, 95% CI 1.86–4.10) [47]. *TP53* mutations, typically found in poorly differentiated and anaplastic thyroid carcinomas, are also linked to shorter OS (HR = 2.87, 95% CI 1.49–5.53) [47]. The presence of these 2nd hit mutations, especially in combination with *BRAF* V600E, identifies a subset of patients with a particularly high risk of recurrence, distant metastasis, and mortality [27, 47]. Their identification supports more aggressive initial management and closer surveillance.

#### *RET/PTC* and other gene rearrangements

*RET/PTC* rearrangements are characteristic of radiation-induced and pediatric PTC but are also found in sporadic adult cases. These gene fusions activate the MAPK pathway, promoting tumorigenesis and are associated with an increased risk of lymph node metastasis and local recurrence in adults [68]. Other actionable rearrangements, such as *NTRK* and *ALK* fusions, though rare, are clinically significant for risk stratification and targeted therapy selection [1, 52].

**Fig. 1:**
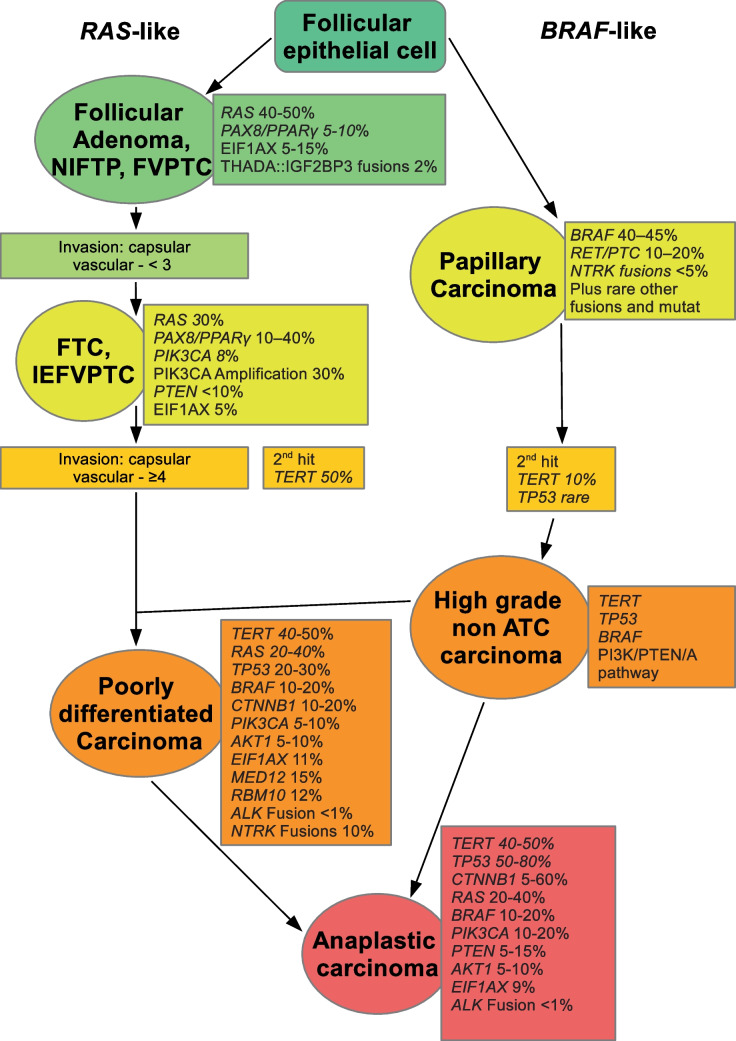
Thyroid tumors evolve along two main axes: RAS-like tumors (including FTA, NIFTP, FVPTC) share RAS mutations and typically have favorable outcomes unless additional mutations occur. BRAF-like tumors (classic PTC and variants) can progress to high-grade and poorly differentiated carcinomas with increasing mutational burden. Both pathways may culminate in aggressive anaplastic thyroid carcinoma characterized by TERT and TP53 mutations, highlighting the interplay of molecular and histologic changes in tumor evolution

Table 2 presents the most important mutations identified in the major types of thyroid carcinomas, summarizing their relative frequencies and clinical significance. It focuses on a selected set of key alterations that are particularly relevant for tumor classification, prognosis, and therapeutic decision-making. Additional mutations not included in this table are illustrated in Fig. 1, providing a more comprehensive overview of the mutational landscape [5, 7, 20, 34, 42, 43, 45, 54, 57].

**Table 2:** Role of selected mutations in different thyroid carcinomas, their frequencies and clinical implications

Mutation/gene	Tumor type(s)	Frequency	Clinical implications
BRAF V600E	Papillary thyroid carcinoma (PTC)	40–45% PTCs	Associated with classic/tall-cell PTC, aggressive features, recurrence risk, prognostic value is limited when considered in isolation
RAS (HRAS, NRAS, KRAS)	Follicular, follicular variant PTC, FTC	13–50% depending on type	Drives follicular-patterned tumors, can indicate dedifferentiation/aggressiveness
RET-PTC rearrangements	Papillary thyroid carcinoma	10–20% PTCs	Early event, associated with radiation exposure, molecular target for cancer treatment
PAX8-PPARγ fusion	Follicular, some FVPTC	10–40%	Diagnostic for FTC/FVPTC, rare in benign lesions, fusion protein may sensitize tumors to PPARγ agonist therapy
TERT promoter	Poorly differentiated, anaplastic, advanced PTC	3–50% (more in aggressive)	Marker of poor prognosis, higher recurrence, mortality
TP53	Anaplastic, poorly differentiated	7–50% (higher in ATC)	Associated with dedifferentiation, poor prognosis, tumor progression
PTEN	Follicular-patterned, indeterminate nodules	Rare	25% risk of malignancy in indeterminate nodules with PTEN mutations
PI3K pathway (e.g., PIK3CA), mutation, and amplification	Poorly differentiated, anaplastic, advanced PTC	Up to 5–20% in ATC/PTC	Indicates poorer prognosis and aggressive biology
NTRK fusions	Papillary carcinoma (esp. children, radiation), poorly differentiated	5–20%	Targetable alterations with specific inhibitors
ALK fusions	Rare PTC subtypes, poorly differentiated	< 1%	Targetable alteration, suggests aggressive potential
RET (MEN2, MTC)	Medullary thyroid carcinoma	40–60% MTC	Prognostic for aggressive disease, risk stratification, direct therapeutic targets, potential hereditary background

## Diagnostic, prognostic, and therapeutic implications

### *RAS*-like and *BRAF*-like tumors—are we ready to move forward?

The landmark publication of The Cancer Genome Atlas (TCGA) data on thyroid cancer revealed that, in well-differentiated carcinomas at least, the mutational burden is surprisingly low. Most tumors could be stratified into two principal molecular subgroups: *RAS*-mutated tumors, which typically display a follicular growth pattern, and *BRAF*-mutated tumors, which are associated with a papillary growth architecture [14]. Over recent years, there has been increasing recognition that such molecular classification provides not only a robust framework for diagnostics but also an essential foundation for patient-centered therapeutic strategies. This perspective has culminated in proposals to eliminate several diagnostically problematic and inconsistently reproducible categories such as the distinctions between follicular thyroid carcinoma (FTC), the follicular variant of papillary thyroid carcinoma (FVPTC), and non-invasive follicular thyroid neoplasm with papillary-like nuclear features (NIFTP) and instead to align diagnostic practice more closely with molecularly defined entities. This shift reflects a broader trend across pathology, where molecular diagnostics have long been integral, as in hematopathology and neuropathology, and more recently in uropathology through the molecular stratification of renal tumor entities in the latest edition of the WHO Classification [5, 6].

### Stepwise dedifferentiation of thyroid carcinomas

The concept of distinct *RAS*-like and *BRAF*-like molecular axes offers a nuanced perspective on the progression of thyroid cancer (Fig. 1). These axes define fundamental tumor entities, with follicular thyroid adenoma (FTA), non-invasive follicular thyroid neoplasm with papillary-like nuclear features (NIFTP), and the follicular variant of papillary thyroid carcinoma (FVPTC) all belonging to the *RAS*-mutated category. Clinically, they lead to similar management strategies—most notably, hemithyroidectomy—with generally excellent outcomes. However, at the molecular level, these tumors remain indistinguishable from true follicular thyroid carcinoma (FTC) or invasive follicular variant papillary thyroid carcinoma (IEFVPTC), provided that no additional mutational events, or “second hits,” have occurred. Histological assessment retains a pivotal role here. The detection of a second hit, such as a *TERT* promoter mutation in up to 50% of cases, significantly worsens prognosis and aligns with the appearance of overt malignancy criteria like extensive capsular and vascular invasion [20, 21]. With advancing tumor progression, molecular evolution can culminate in poorly differentiated and ultimately anaplastic thyroid carcinomas, characterized by more aggressive mutation profiles and clinical behavior.

On the other hand, papillary thyroid carcinomas (PTCs), representing the BRAF-like molecular axis, illustrate a distinct progression pathway. With increasing mutational burden, tumors evolve into a category of high-grade non-anaplastic thyroid carcinomas, further subdivided into high-grade differentiated carcinomas—originating predominantly from the BRAF axis—and poorly differentiated thyroid carcinomas (PDTCs), which may eventually progress to anaplastic thyroid carcinoma (ATC). Evidence supporting this continuum includes observations of residual PTC features within PDTC lesions, characterized by a gradual morphological shift from papillary structures to trabecular, insular, and solid growth patterns, accompanied by a loss of classical PTC nuclear characteristics. Molecularly, a subset of PDTCs harbor BRAF mutations, strengthening the link to this progression pathway.

Since the formal recognition of high-grade differentiated thyroid carcinomas in the latest WHO classification, comprehensive characterization of their mutational spectrum remains incomplete, necessitating further research. Notably, these subgroups commonly exhibit mutations associated with aggressive clinical behavior, such as TERT promoter alterations and TP53 mutations, which correlate with progression to ATC (Fig. 1). This molecular evolution underscores the complexity of thyroid cancer biology and highlights opportunities for future targeted therapeutic interventions.

### Molecular-based diagnosis

The reproducibility of nuclear features in papillary thyroid carcinoma (PTC), together with the determination of the extent of vascular and capsular invasion, has long been a matter of debate among pathologists. This ongoing uncertainty has not only complicated the training of resident physicians, who often struggle to master the complexity of thyroid tumor pathology, but has also resulted in considerable variability among expert observers. The downstream consequence of such diagnostic inconsistency is the production of non-uniform guidelines and, ultimately, the delivery of suboptimal patient care. In order to address these challenges, international experts have recently proposed the establishment of a more robust molecular foundation for diagnostic practice [5].

**Fig. 2:**
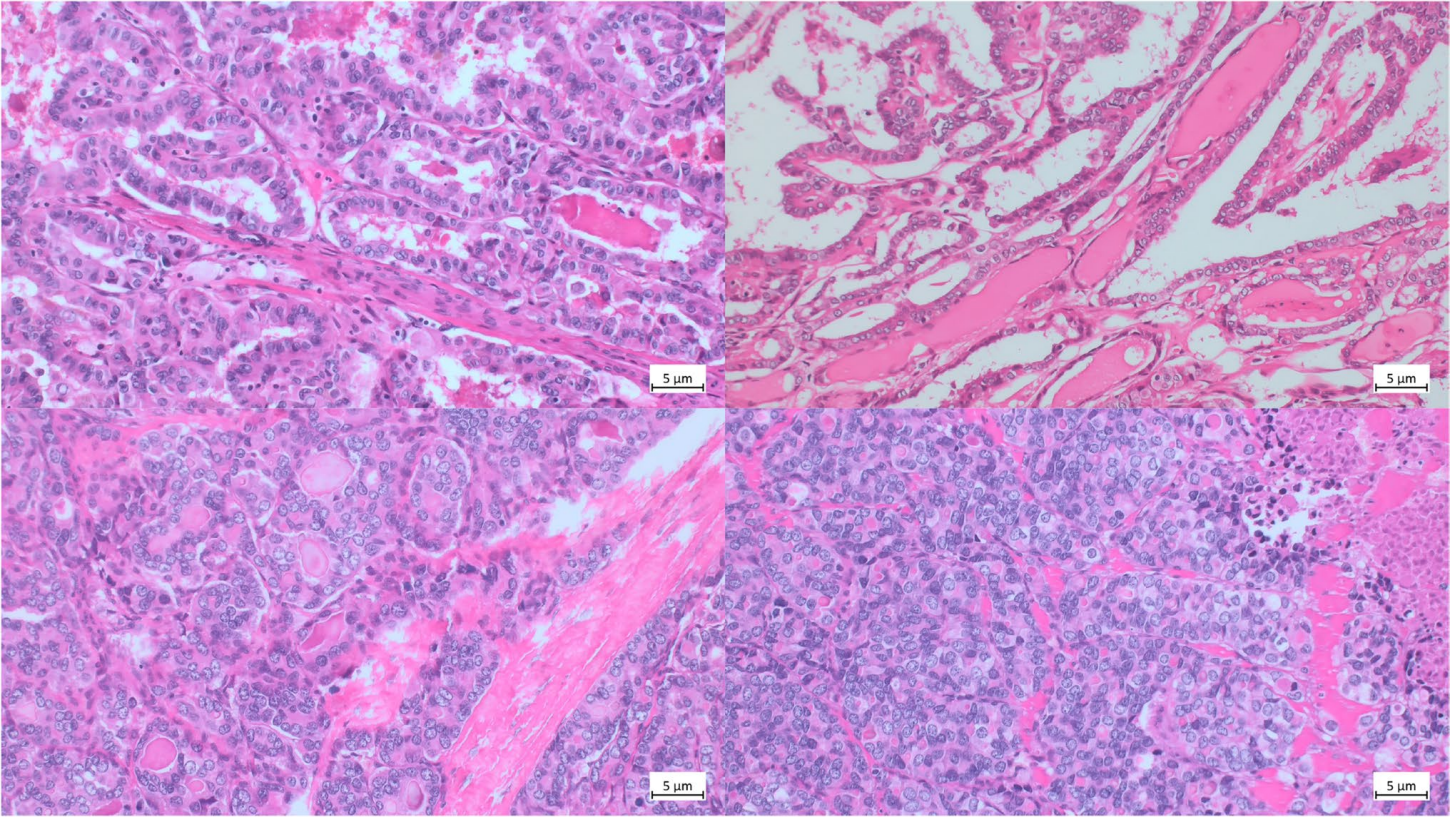
Upper left: Papillary thyroid carcinoma (PTC) without BRAF mutation. Upper right: PTC harboring both BRAF and TERT promoter mutations. Despite being morphologically indistinguishable, the patient without a BRAF mutation demonstrated an excellent clinical outcome, remaining alive after 10 years of follow-up, whereas the patient with both BRAF and TERT mutations succumbed to disease progression. Both lower images are from the same poorly differentiated thyroid carcinoma with a TERT promoter mutation. The possibility of placing such a tumor into that category even if not all morphological criteria are fulfilled would facilitate the diagnostic workup

Figure 2 effectively demonstrates the potential integration of molecular data into the diagnostic schema. Depicted on the left is a papillary thyroid carcinoma (PTC) with a favorable clinical course; this neoplasm harbored a *BRAF* V600E mutation, an alteration also present in the PTC shown in the upper right panel. Nevertheless, the latter case exhibited an additional *TERT* promoter mutation. Despite being morphologically indistinguishable, the tumors exhibited divergent outcomes, with the latter patient succumbing to disease 43 months following the initial diagnosis. Consequently, this neoplasm meets criteria for categorization as a high-grade, non-anaplastic thyroid carcinoma.

The two lower panels illustrate a poorly differentiated thyroid carcinoma from a single case. According to consensus diagnostic frameworks, such an entity requires the presence of at least one among increased mitotic index, convoluted nuclear morphology, or tumor necrosis. In this example, tumor necrosis is discernible in the lower right image, whereas neither elevated mitotic activity nor convoluted nuclei were identified despite extensive histopathological sampling. This scenario recalls those cases in which, notwithstanding a definitive impression of poor differentiation, a formal diagnosis cannot be rendered due to strict adherence to morphological thresholds. The inclusion of robust molecular markers—such as pathogenic TP53 or TERT promoter variants—as ancillary criteria for this diagnosis could enhance diagnostic accuracy and more faithfully represent the underlying tumor biology.

**Fig. 3:**
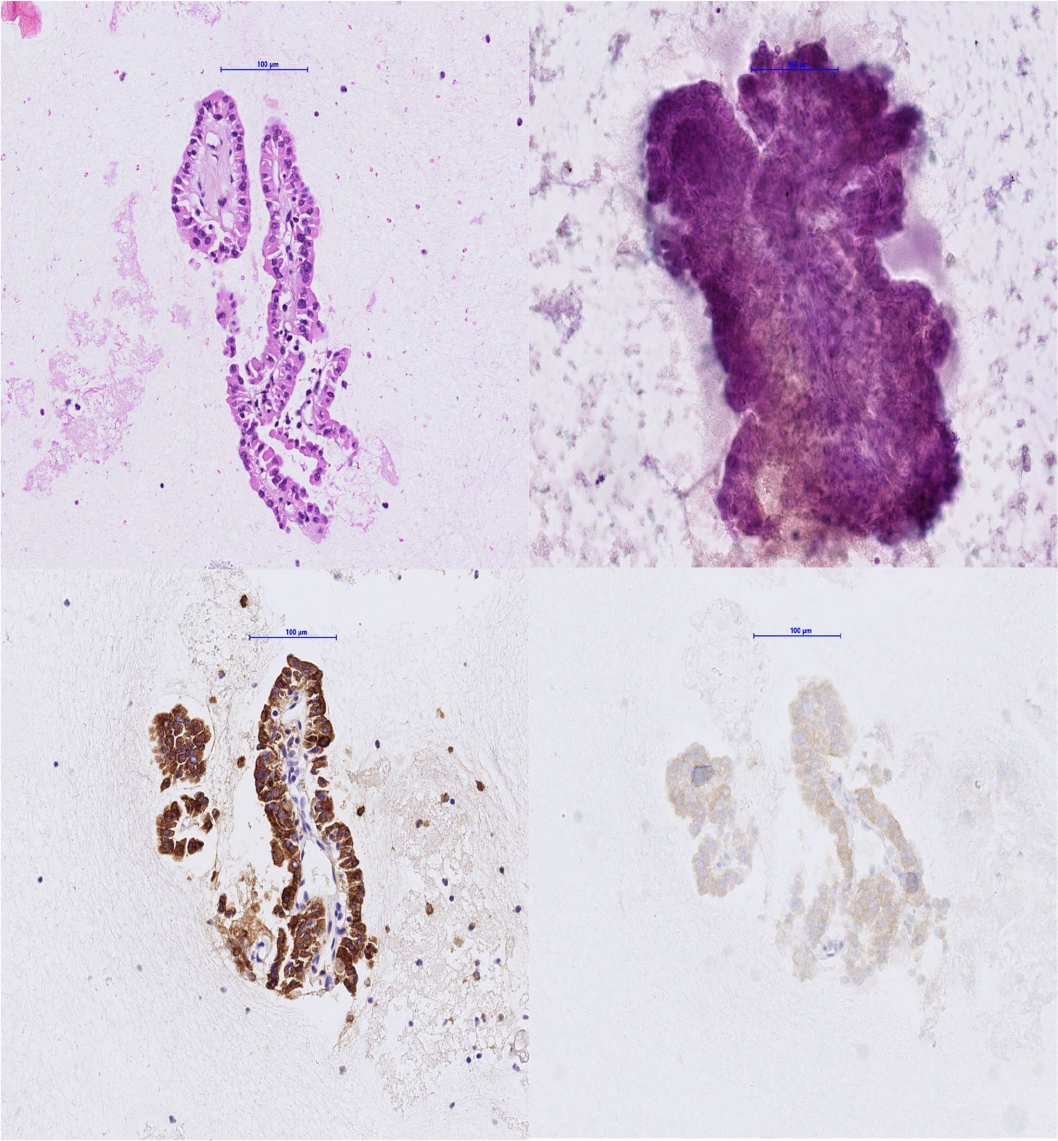
Upper left (cell block HE) and right (Papanicolau): FNA, originally signed out as Bethesda V, suspicious for malignancy. Lower left: immunohistochemistry for thyroglobulin verifies a thyroid origin. Lower right: detection of the BRAF V600E mutation-specific antibody establishes the diagnosis of papillary thyroid carcinoma (PTC)

Although comprehensive molecular testing is not available in all laboratories, the use of mutation-specific antibodies for *BRAF* V600E and *RAS* Q61R, together with immunohistochemical detection of *ALK*, *NTRK*, and *RET* rearrangements, provides practical, accessible, and widely applicable diagnostic strategies that can partially compensate for this limitation [5, 7] (Fig. 3). The systematic integration of such molecular information into thyroid pathology is expected to enhance reproducibility, improve the comparability of clinical studies, support the development of optimized guidelines, and most importantly, result in improved patient outcomes. Thus, the inclusion of molecular data should be regarded not as optional, but as a necessary advancement in the diagnostic approach to thyroid cancer.

### Guiding targeted therapies

#### *BRAF*/*MEK* inhibitors in advanced thyroid cancer

The identification of *BRAF* V600E mutations has direct therapeutic implications, especially in advanced or refractory thyroid cancer. Combination therapy with *BRAF* and *MEK* inhibitors (e.g., dabrafenib and trametinib) has shown significant clinical benefit in *BRAF*-mutated anaplastic thyroid carcinoma, improving progression-free survival and surgical resectability compared to historical standards [52, 68]. This approach is now a standard of care in this aggressive disease subset.

#### *RET* inhibitors and other targeted agents

*RET* fusions, present in a subset of PTC and medullary thyroid carcinomas, are actionable with selective *RET* inhibitors such as selpercatinib and pralsetinib. These agents have demonstrated high response rates and durable disease control in *RET*-altered thyroid cancers. Similarly, *NTRK* and *ALK* inhibitors are available for patients with corresponding gene fusions, further underscoring the necessity of comprehensive molecular profiling in advanced thyroid cancer [24, 52, 68].

#### Role of molecular profiling in clinical trials

Molecular profiling is now fundamental to clinical trial design in thyroid cancer, enabling biomarker-driven patient selection and the development of novel targeted therapies. Trials increasingly stratify patients based on molecular alterations, allowing for more precise evaluation of treatment efficacy and safety [68]. As the repertoire of targeted agents expands, molecular testing will remain essential for guiding enrollment and individualized therapy.

## Limitations and challenges

### Diagnostic accuracy and variability

#### Platform-specific differences in performance

Molecular testing has become integral in the evaluation of indeterminate thyroid nodules, but differences in diagnostic accuracy between platforms remain a challenge. Comparative studies of leading platforms—such as Afirma Genomic Sequencing Classifier (GSC), ThyroSeq v3, and ThyGeNEXT/ThyraMIR—demonstrate similar high sensitivities and negative predictive values (NPVs), but specificities can vary widely. For instance, a meta-analysis revealed that ThyroSeq v3 exhibited a sensitivity of 95.1% and a negative predictive value (NPV) of 92%. However, its specificity was found to be 49.6%, which is lower than the 82% specificity reported in the initial validation studies and comparable to that of Afirma GSC [2, 17]. Other studies confirm that both Afirma GSC and ThyroSeq v3 allow nearly half of patients with indeterminate nodules to avoid diagnostic surgery, but neither platform consistently achieves the high positive predictive value (PPV) and specificity needed to definitively “rule in” malignancy [17, 39, 56]. These differences are influenced by the molecular targets, test methodologies, and population characteristics, complicating direct comparison and clinical decision-making.

#### Partial verification bias and study limitations

A key limitation in the literature is partial verification bias, where only nodules with suspicious or positive molecular results are typically verified by surgery, while benign molecular results are often managed nonoperatively and lack histopathological confirmation [33, 39]. This can inflate sensitivity and NPV, underestimate false negatives, and limit generalizability. Additionally, many studies are retrospective and differ in their handling of borderline lesions such as noninvasive follicular thyroid neoplasm with papillary-like nuclear features (NIFTP), further complicating interpretation and comparison [33, 56]. The evolving nature of molecular platforms and reporting standards also adds complexity to the assessment of diagnostic accuracy.

#### Need for standardized validation protocols

Given these challenges, there is a clear need for standardized validation protocols for molecular testing. Uniform criteria for histopathological endpoints, consistent classification of borderline lesions, and transparent reporting of sensitivity, specificity, PPV, and NPV would facilitate more reliable cross-platform comparisons and improve the evidence base for clinical guidelines [30, 56].

### Economic and accessibility issues

#### Cost-effectiveness and reimbursement

While molecular testing can reduce unnecessary surgeries and associated costs, the high upfront expense and variable reimbursement policies remain barriers to widespread adoption. Cost-effectiveness is highly context-dependent, relying on local healthcare economics, test performance, and prevalence of indeterminate nodules [30, 33]. Some analyses suggest that molecular testing is cost-effective when it prevents surgery in low-risk patients. However, this benefit diminishes when the test has lower specificity or higher costs. Insurance coverage is inconsistent, with some payers requiring specific cytological indications or denying coverage, which can limit patient access and increase out-of-pocket expenses [33].

#### Geographic disparities in test availability

Significant geographic disparities exist in the availability and utilization of molecular testing. In high-income countries, these tests are increasingly integrated into routine care, while in low- and middle-income countries, limited laboratory infrastructure, high costs, and lack of trained personnel restrict access [30, 33]. Even within high-resource settings, disparities persist between urban and rural centers and among different healthcare systems, contributing to unequal patient outcomes.

### Integration into clinical practice

#### Multidisciplinary team approach

Optimal integration of molecular testing requires a multidisciplinary team, including endocrinologists, surgeons, pathologists, radiologists, and genetic counselors. Multidisciplinary tumor boards can help optimize test selection, interpret results, and guide individualized management. However, the lack of standardized guidelines for incorporating molecular results into clinical algorithms leads to variability in practice and can hinder consistent adoption [30, 56].

#### Barriers to adoption in resource-limited settings

In resource-limited settings, barriers include high costs, limited infrastructure, and a lack of clinician awareness or training. The absence of locally validated data and guidelines further impedes adoption. Collaborative research and international partnerships are needed to support capacity building and generate context-specific evidence, ensuring equitable access to the benefits of molecular testing [30, 33].

## Future directions

### Advances in molecular technology

#### Integration of transcriptomic and epigenetic data

Beyond DNA mutation analysis, integration of transcriptomic (RNA expression) and epigenetic data (such as methylation profiles) is emerging as a powerful approach to further refine risk stratification. Early studies suggest that transcriptomic signatures and methylation patterns may distinguish benign from malignant lesions even when DNA mutations are absent, opening new avenues for non-invasive diagnostics and personalized therapy selection [15]. As these technologies mature, their incorporation into routine molecular testing could provide a more comprehensive molecular portrait of each nodule.

### Liquid biopsy and circulating tumor DNA/miRNA

Liquid biopsy—analyzing circulating tumor DNA (ctDNA) or microRNAs (miRNA) in blood—holds promise as a non-invasive tool for diagnosis, monitoring, and early detection of recurrence in thyroid cancer. While still in early clinical development, studies show that ctDNA and miRNA signatures can reflect the mutational landscape of primary tumors and may be particularly useful for patients in whom tissue sampling is challenging [15]. As the sensitivity and specificity of liquid biopsies improve, they could complement or even replace tissue-based molecular testing in certain situations. This would enable real-time disease monitoring and dynamic risk assessment. However, standardized methods and larger studies are needed to confirm their clinical usefulness and overcome current limitations [71].

### Artificial intelligence and risk stratification

#### Digitalization and machine learning algorithms for data interpretation

The complexity and volume of data generated by NGS and multi-omic profiling necessitate advanced computational tools for interpretation. Machine learning algorithms are increasingly being developed to integrate genetic, transcriptomic, and clinical data, improving the accuracy of malignancy prediction and risk stratification [15]. A deep learning algorithm accurately detected the aggressive tall cell subtype of papillary thyroid carcinoma with high sensitivity and specificity in external tissue samples. Its detection correlated with shorter relapse-free survival, showing promise for improving diagnosis and prognosis without retraining [65]. In general, these models have been demonstrated to exhibit the capacity to discern subtle patterns and interactions that may not be identifiable to conventionally trained pathologists. Consequently, these models have the potential to attain a higher level of expertise in the field, thereby supporting more nuanced and individualized clinical decision-making processes [40].

Nevertheless, the current enthusiasm surrounding the application of artificial intelligence (AI) to assist pathologists in achieving accurate diagnoses appears, at present, to be substantially overrated. Despite the abundance of commentary in the literature, the actual primary research foundation remains limited. A search of PubMed (on 22/08/25) identifies only 169 original research papers specifically addressing artificial intelligence in the context of thyroid cancer, in contrast to 1266 secondary publications—including reviews and meta-analyses—on the same topic, representing nearly a tenfold difference. This imbalance highlights the discrepancy between the perceived importance of AI as discussed in the literature and the relatively modest body of original data available to support its clinical implementation.

A major barrier to the global digitalization of pathology laboratories is the lack of harmonized and strict quality control practices both between and within laboratories worldwide, which substantially affects diagnostic reliability and the reproducibility of test results. Moreover, reimbursement for AI-based solutions in pathology remains uncertain and fragmented, with many regions—especially in Europe—lacking established mechanisms to cover AI software expenses, while some systems in the USA offer per-use codes or occasional technology add-on payments amid concerns over overuse. Critically, most currently available AI algorithms in pathology address only one narrow diagnostic task, such as a specific tumor subtype or biomarker identification, which is insufficient for today’s complex, multi-layered diagnostic scenarios that require broader interpretive capability and integration across workflows [31, 55].

### Clinical guidelines and standardization

#### Updates to international guidelines (ETA, ATA, etc.) and recommendations for molecular test utilization

International guidelines, including those from the European Thyroid Association (ETA), American Thyroid Association (ATA), and National Comprehensive Cancer Network (NCCN), are evolving to reflect advances in molecular diagnostics. Recent updates emphasize the importance of validated molecular tests for indeterminate cytology and advanced cancers and recommend their use in conjunction with clinical and radiologic risk factors [9, 15, 42, 45, 61]. The guidelines are also increasingly recognizing the importance of expanded gene panels and the potential of liquid biopsy, though further evidence is needed for widespread adoption.

#### Future research priorities

Future research should focus on large, prospective, multi-institutional studies to validate new molecular markers, transcriptomic and epigenetic signatures, and AI-driven risk models. Efforts are also needed to standardize pre-analytical and analytical procedures, ensure equitable access to advanced diagnostics, and evaluate the cost-effectiveness and real-world impact of these innovations [15]. The integration of liquid biopsy and multi-omic data into clinical practice, as well as the development of global guidelines for molecular test utilization, represents key frontiers for the field.

## Conclusions

Molecular testing has become a transformative tool in the management of indeterminate thyroid nodules, enabling clinicians to reduce unnecessary surgeries and improve risk stratification. Third-generation molecular tests such as Afirma GSC and ThyroSeq v3, which utilize next-generation sequencing and integrated RNA or microRNA analysis, have demonstrated high sensitivity and negative predictive value, allowing clinicians to avoid diagnostic surgery in the majority of patients with indeterminate cytology [3, 17, 19, 69]. These platforms not only help exclude malignancy but also provide information on molecular drivers that can guide the extent of surgery and inform targeted therapeutic options [3].

Despite these advances, challenges persist. There is notable variability in test performance across platforms and clinical settings, and partial verification bias in validation studies can affect the reliability of reported sensitivity and specificity [39]. Furthermore, the lack of standardized protocols and inconsistent classification of borderline lesions such as NIFTP complicates the interpretation and application of molecular results [3, 39]. Economic barriers, disparities in access, and inconsistent insurance coverage further limit the widespread adoption of these technologies, particularly in resource-limited settings [48].

Looking ahead, the future of molecular testing in thyroid pathology is promising. Ongoing advances in next-generation sequencing, liquid biopsy, and integration of multi-omic data are expected to further refine diagnostic precision and enable dynamic, personalized risk assessment [3, 56]. Updates to international guidelines and consensus recommendations will be crucial for standardizing the use of molecular diagnostics and ensuring their evidence-based integration into clinical practice [3, 61].

A call to action is warranted: future research should prioritize large-scale, prospective studies with standardized protocols to validate emerging biomarkers and technologies. Efforts to improve cost-effectiveness, accessibility, and equitable integration into diverse healthcare systems are essential. Ultimately, molecular diagnostics should be embraced as a complementary tool within multidisciplinary care, optimizing outcomes for patients with thyroid nodules and cancer.
